# Surface Finishing and Coating Parameters Impact on Additively Manufactured Binder-Jetted Steel–Bronze Composites

**DOI:** 10.3390/ma17030598

**Published:** 2024-01-26

**Authors:** Andrew C. Grizzle, Amy Elliott, Kate L. Klein, Pawan Tyagi

**Affiliations:** 1 Department of Mechanical Engineering, University of the District of Columbia, Washington, DC 20008, USA; andrew.grizzle@udc.edu (A.C.G.);; 2 Oak Ridge National Laboratory, Knoxville, TN 37830, USA; elliottam@ornl.gov

**Keywords:** binder jetting, nickel plating, surface roughness, additive manufacturing, rapid prototyping, post-processing

## Abstract

In this paper, electroless nickel plating is explored for the protection of binder-jetting-based additively manufactured (AM) composite materials. Electroless nickel plating was attempted on binder-jetted composites composed of stainless steel and bronze, resulting in differences in the physicochemical properties. We investigated the impact of surface finishing, plating solution chemistry, and plating parameters to attain a wide range of surface morphologies and roughness levels. We employed the Keyence microscope to quantitatively evaluate dramatically different surface properties before and after the coating of AM composites. Scanning electron microscopy revealed a wide range of microstructural properties in relation to each combination of surface finishing and coating parameters. We studied chempolishing, plasma cleaning, and organic cleaning as the surface preparation methods prior to coating. We found that surface preparation dictated the surface roughness. Taguchi statistical analysis was performed to investigate the relative strength of experimental factors and interconnectedness among process parameters to attain optimum coating qualities. The quantitative impacts of phosphorous level, temperature, surface preparation, and time factor on the roughness of the nickel-plated surface were 17.95%, 8.2%, 50.02%, and 13.21%, respectively. On the other hand, the quantitative impacts of phosphorous level, temperature, surface preparation, and time factor on the thickness of nickel plating were 35.12%, 41.40%, 3.87%, and 18.24%, respectively. The optimum combination of the factors’ level projected the lowest roughness of Ra at 7.76 µm. The optimum combination of the factors’ level projected the maximum achievable thickness of ~149 µm. This paper provides insights into coating process for overcoming the sensitivity of AM composites in hazardous application spaces via robust coating.

## 1. Introduction

Additive manufacturing (AM) technologies are catalyzing an industrial revolution by impacting the innovation and manufacturing processes in critical fields such as the aerospace, biomedical, and automotive industries [1,2,3,4,5,6]. AM components can produced by utilizing a vast list of materials, including metals, polymers, and composites, with a high degree of geometric complexity [7,8]. AM is hence producing designs and functionalities that go beyond the imagination of innovators and technology leaders [9]. There are seven key additive manufacturing technologies: binder jetting, directed energy deposition (DED), material extrusion, material jetting, powder bed fusion (PBF), sheet lamination, and vat photopolymerization. The binder-jetted composite (BJC) fabrication process used in this study has several advantages over other forms of AM depending on the requirements, particularly in terms of the significantly improved speed and resolution. Binder jetting works by spreading a layer of powder and binding the shape of the layer with an inkjet printhead and binding ink. Once the part is bound, it is removed from the powder bed and consolidated via sintering or infiltration. Infiltration is a strategic processing choice for binder jetting to avoid the shrinkage and distortion that comes with sintering. BJCs have isotropic microstructure properties and no residual thermal stress [10]. Like traditional mechanical parts, AM components can be sensitive to wear, corrosion, fatigue, stress, and shear [11]. Ideally, additive manufacturing (AM) components should possess durability, longevity, and resistance to corrosion. However, achieving all the required mechanical attributes with a singular material or technique poses challenges. Depending on the specific AM process, certain components might exhibit impressive surface properties but are vulnerable to stress and loads. Conversely, other components might withstand deformation but are prone to corrosion. Typically, corrosion and cracks are closely linked to the surface quality of the produced part [12]. The likelihood of failure is significant when components have a poor surface finish [13]. In such cases, classic surface strengthening and corrosion prevention procedures such as heat and chemical treatment, spray coating, and electrolytic and electroless plating can be highly effective [14,15]. Importantly, the outcomes of these procedures may be uncertain when dealing with a component constructed from a mix of metals within a non-uniform matrix or pattern. Specifically, binder-jetted composites (BJCs), which involve at least one hard and one soft material, might exhibit greater susceptibility to corrosion and wear compared to conventional components made from a single alloy, using processes like laser sintering or other high-temperature methods. In this current study, we have implemented surface-finishing techniques to enhance the surface quality of BJCs, with the intention of subsequently achieving a high-quality coating. The application of a nickel coating is anticipated to provide protection to BJCs with diverse components that possess varying physicochemical properties in their as-produced state. Employing multi-variable analysis in conjunction with the Taguchi design of experiments, we aim to explore the impacts of different coating parameters in achieving a smooth, electroless nickel coating thickness.

## 2. Methods and Materials

The BJC (binder-jetted component) employed in our investigation consists of a composition of 60% 420 stainless steel and 40% bronze infiltration. The manufacturing process details are provided elsewhere [16]. We constructed an empirical model with the objective of achieving a smooth surface morphology for several-micron-thick nickel depositions on nine binder-jetted components made of 420 stainless steel and bronze. The experimental design for these nine samples was based on the Taguchi Design of Experiment, allowing the exploration of multiple variables and their levels in fewer experiments compared to a plan where one variable is altered at a time [17].

We employed Taguchi’s parameter design approach to investigate the impact of various electroless Ni deposition process parameters on the roughness and thickness of the deposition. Our emphasis was on Ni thickness due to the unknown correlation between nickel growth rates and different process parameters. Specifically, we utilized Taguchi L9(3^4) orthogonal arrays designed to explore nonlinear relationships among four factors, each with three discrete levels. An empirical model was developed for these four process parameters. The first factor considered was the phosphorus content in the plating solution. We utilized pre-prepared nickel solutions with high (10–13%), medium (6–9%), and low (1–4%) phosphorus contents. The second factor was the solution temperature. Recommended temperatures were 90 °C for low- and mid-phosphorus Ni solutions and 85 °C for high-phosphorous Ni plating solutions. We set low, medium, and high levels by maintaining the deposition temperature at RT −10 °C, RT, and RT +10 °C, respectively. The third factor involved the surface preparation of BJC, with three levels: organic-solution-cleaned (OC), plasma-cleaned (PC), and chempolished (CP) preparations. The fourth factor considered the time required to deposit varying thicknesses. We determined the time for depositing 20, 30, and 40 µm thicknesses based on the data provided by the manufacturer’s datasheet. The estimated deposition time was derived from the plating rate at RT for low- and mid-phosphorous (17 microns/h) and high-phosphorous (12 microns/h) Ni solutions. The complete list of factors and associated levels is detailed in Table 1.

The primary focus of this investigation was BJC’s surface preparation. Organic cleaning (OC) involves a sequential soaking process in acetone, isopropanol alcohol, and deionized water, each for a duration of 1 min. During the OC treatment, BJC underwent ultrasonication in an ultrasonic agitator. The final step of the OC treatment involved a thorough rinse in deionized water. The second surface preparation method employed was chempolishing (CP). In the chempolishing process, BJC samples underwent polishing for 30 min at 75 °C in a DS-9-314 chemical solution. This proprietary chempolishing solution, provided by Dubois Chemicals^®^ in Vancouver, BC, Canada, comprises 10–30% phosphoric acid, 1–10% hydrochloric acid, 1–10% nitric acid, and 1–10% proprietary surfactants. The third surface treatment involved plasma cleaning (PC), employing a 10 min argon plasma treatment. The plasma was generated with 100 W RF power, at a 30 SCCM Ar flow rate, and a pressure of 320 mTorr to isotropically etch BJ samples. Additionally, we defined the exposed area on BJC for electroless nickel coating. A thick layer of Shipley 1813 positive-tone photoresist was applied to establish a consistent surface area on the BJCs following surface preparation. The electroless nickel plating bath solution, provided by Plating International^®^, Franklin Park, IL, USA, utilized a ‘One Plate LP’ process with self-regulating pH and variable phosphorous content. Three distinct plating solutions with low, medium, and high phosphorus levels were employed in each of the nine experiments. The electroless plating solution was thoroughly mixed using a magnetic stirrer rotating at 300 RPM.

Derived from the selected factors and levels outlined in Table 1, we formulated a series of nine experiments using the L9 Taguchi Design of Experiment. Each experiment was assigned a distinctive ID corresponding to the surface preparation method employed before the electroless nickel coating process, as detailed in Table 2.

We produced one sample for each experiment run, which are listed in Table 2. The analysis encompasses the nine samples detailed in Table 2, examining their surface roughness and morphology both before and after the electroless nickel coating process. At least five measurements of roughness and film thickness were accomplished for the analysis. Surface roughness was measured using a Keyence microscope, Itasca, IL, USA, while microstructural and elemental analysis were performed using the Phenom XL-30 SEM, Alexandria, VA, USA. Additionally, various measurements including reflectance, contact angle, and scratch tests were conducted to assess diverse coating properties. However, this paper predominantly concentrates on the surface roughness and thickness of the electroless coating. Subsequently, a Taguchi design of experiment-specific statistical analysis was employed to explore the influence of each factor and their respective levels on different properties of the electroless nickel coating. We analyzed raw data to learn the effect of different levels of each factor and levels by using the statistical mathematical formalism associated with Taguchi method [18].

## 3. Results and Discussion

### Surface Roughness

**Figure 1:**
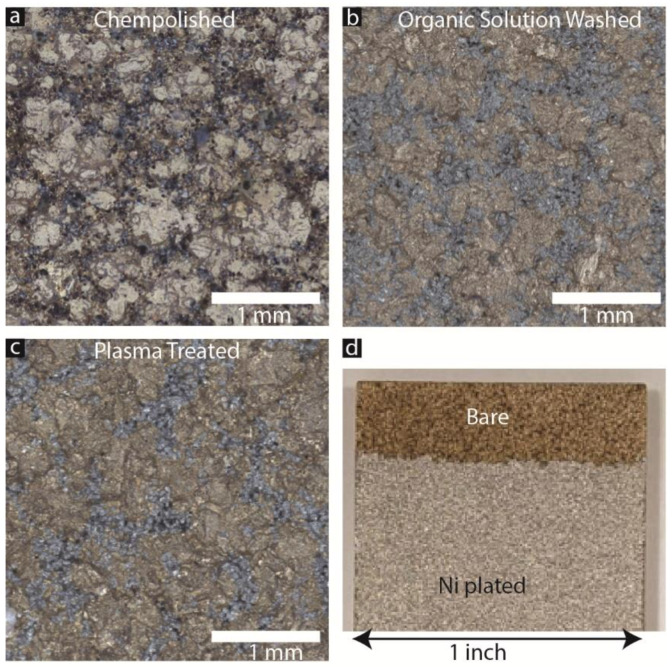
Image of binder-jetted substrates after surface preparation. BJC after (**a**) chempolishing, (**b**) organically cleaning, and (**c**) plasma cleaning. (**d**) Representative sample showing a partly protected bare section of each sample while doing electroless nickel coating on the rest of the surface.

Preparing the surfaces of composite samples is a critical step in the nickel (Ni) plating process, potentially influencing surface morphology, energy levels, and overall plating quality. We utilized the surface preparation method described in the previous section to qualitatively assess surface characteristics before nickel deposition. Our findings revealed that chempolished sample surfaces (Figure 1a) exhibited more pitting and material removal, indicating a rougher and somewhat more uniform material. To gauge apparent changes in surface color and texture, we used an organic-solution-cleaned sample (Figure 1b) as a reference. Plasma etching, illustrated in Figure 1c, unveiled a surface akin to that of the organic solution, albeit with a slightly reduced surface roughness compared to the plasma-etched sample. Recognizing the significant impact of surface preparation on roughness, the subsequent experiment aimed to quantitatively measure these changes both before and after the application of electroless Ni coating. Each of the nine samples has individual surface roughness and properties. Hence, we studied each of the nine samples before and after the surface preparation and electroless nickel coating process. Hence, an uncoated portion of each sample was a reference point for itself. Figure 1d shows a typical sample that has a bare and plated surface.

**Figure 2:**
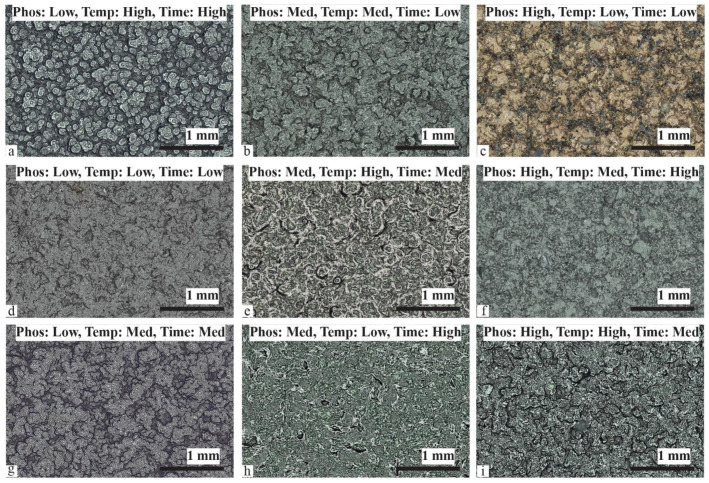
Images of Ni-plated BJC based on nine different experiments. Chempolished samples nickel coated (**a**) with low P, high temperature, and longtime duration; (**b**) with medium P, medium temperature, and smallest time duration; (**c**) with high P, low temperature, and smallest time duration. Organically cleaned nickel-coated BJC sample with (**d**) low P, low temperature, and smallest coating duration; (**e**) medium P, high temperature, and medium timing; and (**f**) high P, medium temperature, and the longest deposition time. Plasma-cleaned samples were nickel coated with (**g**) low P, medium temperature, and medium deposition duration, (**h**) medium phosphorous, low temperature, and the longest deposition period; and (**i**) high P, high temperature, and medium deposition duration.

We performed a qualitative examination of morphological differences in Ni coatings on composite substrates, as depicted in Figure 2.

The images are annotated based on the experimental runs outlined in Table 2. The results are organized by the surface finishing method. The top, middle, and bottom rows represent chempolished, organic, and plasma-cleaned groups, respectively. Upon visual inspection, noticeable distinctions in the reflective and homogeneous characteristics of the coatings are apparent. Notably, there are clear visual variations in plating texture observed among the Ni-plated groups. Particularly intriguing behavior was observed in the case of chempolished BJC. Low P, high temperature, and long deposition duration produced the high granularity on nickel coating (Figure 2a). With medium P, medium temperature, and the smallest deposition duration, Ni deposition was observed (Figure 2b). Interestingly, negligible deposition occurred for low temperature, small duration, and high P content (Figure 2c). It is noteworthy that small deposition duration and low temperature were able to yield significant deposition on the organically cleaned sample (Figure 2d). Hence, the results in Figure 2c are due to either the P content or the surface finishing effect. The organically cleaned sample yielded nickel plating for medium P (Figure 2e) and high P (Figure 2f) content. However, the morphology of the organically cleaned sample was dramatically different (Figure 2e,f). After undergoing plasma cleaning, BJC exhibited distinct surface morphologies following nickel plating, as depicted in Figure 2g–i. Films displaying increased roughness were produced with lower phosphorus content, moderate temperature, and a moderate plating duration. Interestingly, smoother morphologies were attained with medium phosphorus content, lower temperature, and an extended plating duration. The result depicted in Figure 2h closely mirrored the outcomes associated with higher phosphorus content, moderate temperature, and an extended plating duration. Plating under conditions of elevated phosphorus content, increased temperature, and a moderate deposition time led to a rough morphology (Figure 2i).

The chempolished group (Figure 2c) showed no Ni plating, while the organically cleaned sample showed the smoothest morphology (Figure 2d,f). The stark difference in Ni thickness and quality may be a result of the combination of process parameters. Conversely, we see that, among the three surface preparation groups, medium phosphorous levels (Figure 2b,e,h) show relatively consistent Ni plating shininess and homogeneity in comparison to the low and high phosphorous level groups. All the nickel coatings are expected to perform very differently when subjected to a challenging application environment. To gain deeper insights, we have conducted SEM studies at higher magnification. The following section looks at these results at the microscopic scale.

**Figure 3:**
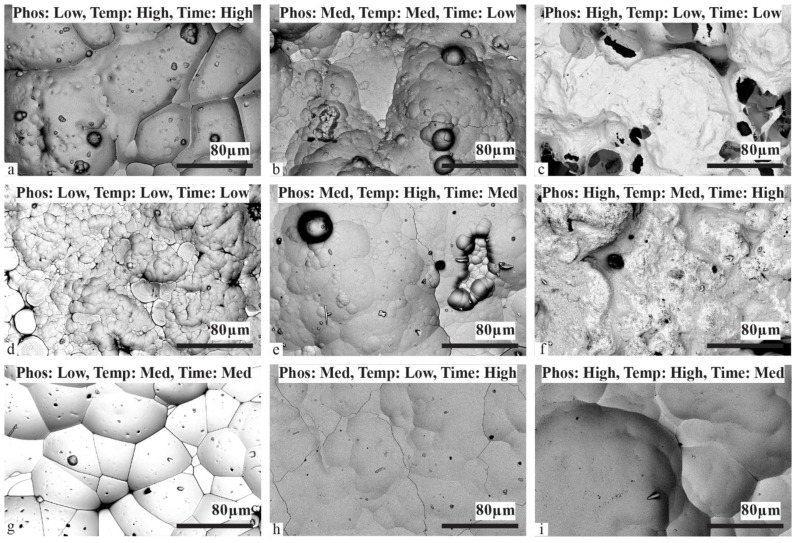
SEM image of Ni-plated composite surfaces: Chempolished BJE treated with nickel plating conditions as per DOE experimental plan (**a**) 1, (**b**) 2, and (**c**) 3. Organically cleaned BJE treated with nickel plating conditions as per DOE experimental plan (**d**) 4, (**e**) 5, and (**f**) 6. Plasma-cleaned BJE treated with nickel plating conditions as per DOE experimental plan (**g**) 7, (**h**) 8, and (**i**) 9.

Chempolished BJE appears to have a significantly rough texture in general. CP-1 sample produced the continuous film with varied granular scale (Figure 3a). CP-2 BJE produced grains of the order of 10 µm (Figure 2b). However, the CP-3 BJE sample had a very rough surface (Figure 3c). High roughness is consistent with discontinuous Ni deposition as shown in Figure 2c. BJE after chempolishing were also very rough (Figure 1c). However, organically cleaned samples showed much smoother Ni plating morphologies. Low P and temperature with the small duration of deposition produced much smaller granularity (Figure 3d). On OC-2 BJE, medium-P, high temperature, and medium deposition duration produced bigger grain (Figure 3e). However, OC-3, where high P, medium temperature, and long deposition duration were employed, the BJE produced a fine granular structure (Figure 3f). Similarly, we studied microscopic details of the plasma-cleaned samples. The PC-1 sample, produced with low P plating solution, medium temperature, and medium plating duration, yielded a peculiar closely packed pattern of granules (Figure 3g). Interestingly, the boundaries between granules were very sharp. PC-2 BJE yielded relatively smooth morphologies (Figure 3h). However, PC-3 BJE showed high granularity with significantly high variation in features height (Figure 3i). An SEM study has revealed micro-scale deposition features based on the electroless nickel plating process parameters (Figure 3). We found that, in general, the Ni coatings had a cauliflower-like texture. Of the surface preparation groups, plasma etching (Figure 3g–i) had more consistent features with medium-size granules. The organic cleaning group (Figure 3d–f) had relatively more minor cells but varying perceived Ni coating roughness and thickness. The chempolished group demonstrated the most variation of the cauliflower-like cell structures in terms of cell size and coating thickness (Figure 3a–c). The samples with the least apparent surface roughness are the medium phosphorous level groups (Figure 3b,e,h). Referring to Table 2, the implication is that the medium phosphorous level Ni coating solution provides a visually better coating quality despite the variation of the other three process parameters.

**Figure 4:**
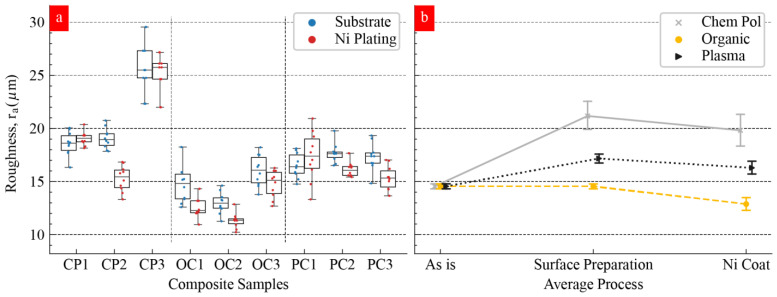
(**a**) Distribution of roughness measurements on samples before and after applying Ni plating. (**b**) Surface preparation effect on roughness.

To quantitatively determine the surface roughness of Ni coating, we conducted surface roughness measurements on each sample before and after surface preparation and Ni deposition. Each of the nine samples has individual surface roughness and properties. Hence, we preferred to study each of the nine samples before and after the surface preparation and electroless nickel coating process. Hence, each sample was a reference point for itself. In Figure 4, we show the roughness data for each sample in different stages. Figure 4a shows the *Ra* roughness value of each of the nine samples before and after Ni deposition. The main aim of Figure 4 is to compare the distribution of the roughness data based on the surface preparation methods (*x*-axis) and Ni coating state (color legend). CP-3 BJE produced the highest roughness, and this measurement is consistent with the SEM study (Figure 2c and Figure 3c). The SEM study showed persistent porosities and pitting on the Ni-coated sample (Figure 3c). We observed negligible Ni deposition on CP3, and the microstructure resembled the microstructure obtained after chempolishing. CP3 yielded the highest ~25 µm Ra roughness (Figure 4a). It is noteworthy that we did not impact the bulk properties, such as porosities, of the samples and all the changes are limited to the outer surface. OC 1–3 produced the least surface roughness after the Ni coating on BJEs. It is noteworthy that the least roughness on BJE after Ni coating was smaller than that observed on an organically cleaned surface. It seems significant that Ni deposition occurred on OC samples that reduced the difference between the hills and valleys present on the OC samples before Ni deposition.

Figure 4b summarizes the roughness effect based on the cleaning method (legend) and the Ni coating effect. We found that, with the exception of the organic cleaned group (OC-1:3), surface preparation generally increases substrate roughness, with the chempolished group (CP-1:3) creating significantly higher roughness values. As expected, the Ni deposition process generally reduced surface roughness, with the medium phosphorus level group (CP-2, OC-2, and PC-2) showing a more consistent reduction in relation to low and high phosphorus levels. We found that CP-2, OC-2, and PC-3 produced the lowest levels of surface roughness within the surface preparation group.

**Figure 5:**
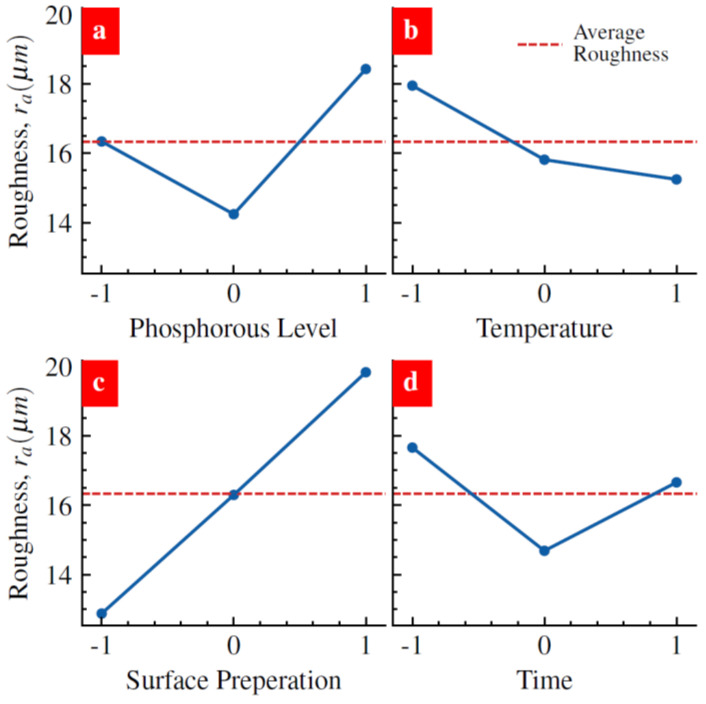
Impact of levels of (**a**) phosphorous, (**b**) temperature, (**c**) surface preparation, and (**d**) time factors on surface roughness. Blue lines connecting the data points are only guide to eye.

We analyzed the roughness data to understand the impact of the individual factors. The emphasis on surface roughness is due to the impact of surface finishing on the properties of nickel-plated AM components. The mean response refers to the average value of the performance characteristics for each of the four parameters at different levels (Figure 5), which, when studied together, represents the main effects of the process parameters. To quantify these characteristics, we ran a main effect study to understand how the changing Ni coating process parameters affect surface roughness. These values are illustrated in Figure 5a–d. The phosphorous levels in the nickel plating solution produced no linear impacts (Figure 5a). The low phosphorous levels did not impact the initial surface roughness level (Figure 5a). Interestingly, the medium phosphorous level improved the surface roughness, and the high phosphorous level worsened the surface roughness (Figure 5a). The raw data in Figure 4b is consistent with the analysis data in Figure 5a. Three temperature levels during Ni plating also produced a nonlinear impact (Figure 5b). Low temperatures produced rougher Ni film as compared to the other two temperature levels (Figure 5b). The surface preparation methods appear to create the most significant impact (Figure 5c). Organic cleaning helped to reduce the surface roughness. However, chempolishing worsens the surface roughness (Figure 5c). These analysis results are consistent with the SEM images showing the surface morphologies (Figure 2, Figure 3 and Figure 4). Interestingly, the effect of the time factor on roughness also followed a nonlinear trend (Figure 5d). It is noteworthy that the plating temperature set above the recommended temperature led to smoother Ni film. We see that surface preparation has the most significant influence on the roughness value around the average. As surface preparation varies from organic solution to chempolishing, the mean response increases by approximately 7 µm. Surprisingly, Ni coating temperature had a negligible impact on the roughness value. In the present case, lower temperatures produce a ~3 µm decrease in surface roughness. For both the phosphorous level and the time parameters, their medium mean response provides the lowest surface values.

Additionally, to assess the significance of these process parameters on roughness, we conducted an analysis of variance (ANOVA). The ANOVA results for the raw data on roughness are presented in Table 3. The last column, indicating the percentage influence (*P*%), reveals the extent of impact attributed to each factor. It is evident that surface preparation exerted the most substantial influence on the roughness of the Ni-plated BJEs. These quantitative findings align with the reported surface morphology in Figure 2a. The phosphorus level emerged as the second most significant factor (Table 3). Notably, nickel plating time exhibited the least influence, falling below the combined error of this study. The ANOVA analysis quantitatively underscores that, for achieving the smoothest surface roughness on Ni-coated BJEs, selecting an appropriate surface finishing method is paramount.

We also conducted a Taguchi design analysis to determine the combination of the process parameters for achieving the lowest surface roughness achievable with the given process parameter space. The promising values of different parameters for achieving the lowest roughness are tabulated in Table 4. According to the analysis, medium-level phosphorus, high temperature, low time, and surface preparation with organic solution are projected to produce the lowest value of Ni coating roughness. The optimal combination of process parameters reduces the average roughness value from 16.33 µm to 7.76 µm, reducing surface roughness by 52.5%. In the recommended combination of parameters, the surface preparation type matches the observation of obtaining the smoothest surface morphology after OC treatment (Figure 2). Also, low deposition time is justifiable because thick films generally start showing rougher morphologies due to growth dynamics and structural stresses.

**Figure 6:**
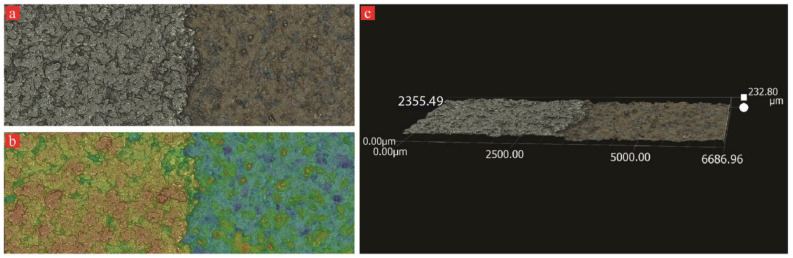
Keyence microscope 3D mapping of representative Ni-plated composite surface: (**a**) raw image obtained from the microscope, (**b**) color-coded image for differentiating between nickel coating and base material, and (**c**) 3D perspective image of step used for nickel coating thickness.

**Figure 7:**
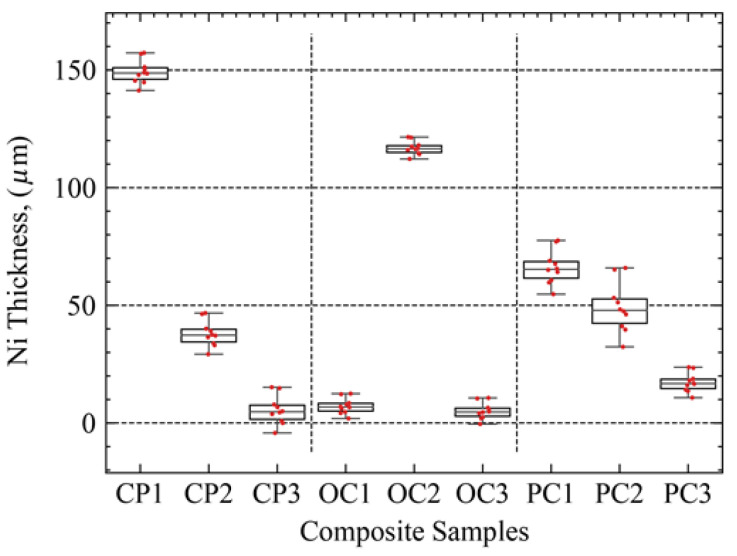
Ni plating thickness on nine samples produced in this study.

To estimate the process parameters for achieving the highest deposition thickness, we measured the thickness of the Ni coating for each sample. A Keyence light microscope was used to create a high-resolution 3D surface map that overlaps the extent of the Ni coating and the surface of the substrate (Figure 6a). For the measurement of the step height between Ni coating and the surface of BJE, instrument analysis software was utilized (Figure 6b). The 3D perspective image suggested that we have attained significant Ni deposition (Figure 6c). The resulting height and location data are illustrated in Figure 6b,c. Horizontal line profiles were used to evaluate height differences between Ni plating and substrate surface for each sample, and these measurements are illustrated in Figure 7. We found that the CP group had the highest and lowest deposition rates in all samples, as shown in Figure 7. To a lesser extent, the coating thickness varied similarly in the OC and PC groups, with the PC group having relatively low variation.

**Figure 8:**
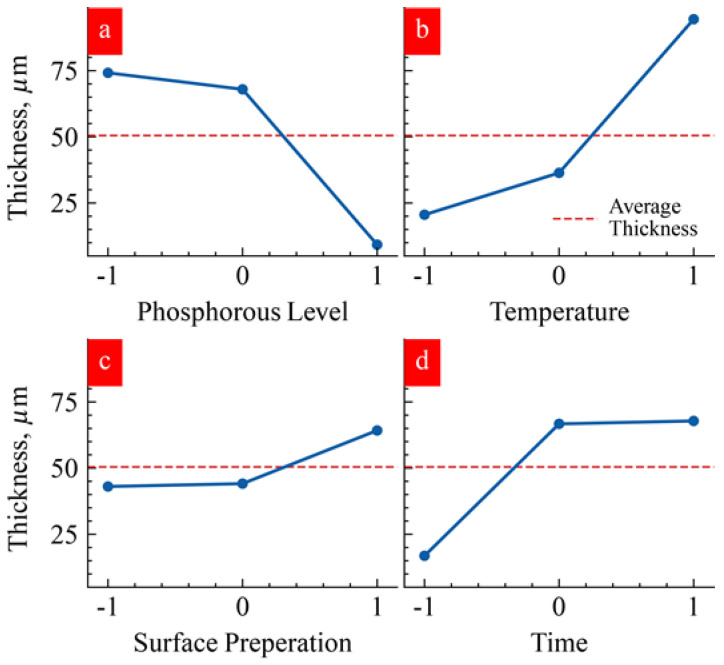
Taguchi analysis illustrating the effect of various levels of (**a**) phosphorous, (**b**) temperature, (**c**) surface preparation, and (**d**) time factors on Ni thickness. Blue lines connecting the data points are only guide to eye.

We conducted a statistical Taguchi analysis to understand the effect of individual levels of four factors on the thickness of the electroless coating. The graphs of the main effects in Figure 8 illustrate the thickness of Ni deposition as a function of the levels of the process parameters. Low and medium phosphorous levels produced a similar influence (Figure 8a). However, high phosphorus levels negatively impacted the thickness of Ni plating (Figure 8a). The mechanism behind the phosphorous content’s impact on plating thickness is not completely clear. However, low and medium phosphorous content appears to yield lower grain growth size than grain growth caused by the high phosphorous content (Figure 3). Smaller grain growth is due to the dominance of higher nucleation rate compared to the growth rate of the individual nuclei. High temperature was much more influential in giving higher thickness (Figure 8b). The effect of temperature is consistent with the fact that growth kinetics accelerate with increasing temperature. Surprisingly, surface preparation levels did not impact Ni thickness (Figure 8c). It is noteworthy that substrate preparation with organic cleaning (−1) and plasma cleaning (0) process produced a similar effect on nickel thickness; this result can be explained based on the similarity in surface morphology and chemistry after the organic cleaning and plasma cleaning process as shown in Figure 1b,c. Chempolished samples, producing the highest surface roughness of Ra ≈ 18–26 µm, produced the highest thickness growth. Notably, chempolishing generally etches away the iron particles, leaving a bronze-rich phase. Interestingly, deposition time showed a nonlinear trend (Figure 8d). Low deposition time produced the least impact on thickness. However, medium and high deposition times resulted from the same effect (Figure 8d). It appears that, after reaching a specific thickness, deposition did not proceed.

We conducted an ANOVA analysis to assess the percentage impact of each factor on Ni thickness, as detailed in Table 5. The results indicated that the temperature and phosphorus level of the Ni plating solution exerted the most substantial influence on the thickness change of the coating. The heightened impact of temperature on Ni thickness is attributed to the exponential dependence of any catalytic reaction on temperature. Additionally, the growth rate is contingent on the chemical composition of the bath, making phosphorus content pivotal in governing the growth rate. Notably, an increase in phosphorus level correlated with a general reduction in the deposition rate, while an increase in solution temperature correlated with a general increase in the deposition rate. In contrast, the surface preparation of the substrates had a relatively minor impact on the deposition rate (Table 5).

Using the Taguchi statistical analysis [17,19], we determined the optimal combination of process parameters within the study space for the highest deposition rate/thickness and tabulated the results in Table 6. We saw that the highest thickness of Ni plating was expected when the Ni plating used a low-level phosphorus solution at high temperatures and with more time. Furthermore, chempolishing was recommended as surface preparation; however, as shown in Figure 8 and Table 6, the surface preparation process parameter had a statistically insignificant effect on the thickness of the Ni plating.

## 4. Conclusions

Nine electroless nickel depositions, employing varied process parameters on binder-jetted composite materials, were characterized and studied to achieve coatings that are both smooth and thick. The key highlights of this paper are presented here.

The chempolishing treatment resulted in the selective etching of the exposed stainless phase near the surface regions, leaving bronze-rich phases on the surface. Our study emphasizes the need for careful consideration when selecting surface finishing methods for BJC to prevent complications arising from undesired surface chemistry and morphology post-surface finishing. Optical and SEM images revealed that samples coated with medium phosphorous levels in electroless Ni solutions consistently produced better coverage and shine in the coatings. Within the surface preparation groups, the plasma treatment exhibited the most visually consistent reflective Ni coating results. In contrast, the chempolishing group resulted in increased surface roughness and decreased surface energy. Generally, the addition of Ni-P coatings contributed to a reduction in roughness. The main effect results indicate that, for the specified parameters, the optimal levels for minimizing surface roughness are observed with medium phosphorus levels, high solution temperatures, organic cleaning processes, and low coating times. The study also revealed that the impact of four factors on nickel-plating thickness varied significantly. Those aiming for a thick Ni film should focus on the phosphorous level, as time and temperature nonlinearly influence Ni thickness. The optimal values for parameters differ when seeking minimal roughness versus maximum coating thickness. This observation suggests that obtaining the same parameter set for multiple desired properties is unlikely. In future work, we intend to explore the impact of the discussed parameters on the physical, chemical, and mechanical properties of the film. Future studies will focus on utilizing XRD for the evaluation of the crystallinity of the electroless nickel-coated films to gain additional insights about the process parameters’ impact on desired properties. In future work, we also plan to explore the correlation between experimental parameters and the mechanical properties of electroless nickel plating.

## Figures and Tables

**Table 1:** Process parameters and their values at different levels.

Factors	Level −1	Level 0	Level 1
Phosphorous Level (%)	1–4	6–9	10–13
Temperature (°C)	RT − 10	RT	RT + 10
Surface Preparation	Organic	Plasma	Chempolished
Target thickness	20 µm	30 µm	40 µm

**Table 2:** The L9 orthogonal array showing values of four factors: phosphorous level (P), temperature (Temp.), surface preparation (Prep.), and target thickness (µm).

Exp. Run	P (%)	Temp. (°C)	Prep.	Thickness (µm)	ID
1	1–4	80	Organic	20	OC1
2	1–4	90	Plasma	30	PC1
3	1–4	100	Chempolish	40	CP1
4	6–9	80	Plasma	40	PC2
5	6–9	90	Chempolish	20	CP2
6	6–9	100	Organic	30	OC2
7	10–13	75	Chempolish	20	CP3
8	10–13	85	Organic	40	OC3
9	10–13	95	Plasma	30	PC3

**Table 3:** ANOVA of roughness data.

Factor	DOF	*SS*	*V*	F-Ratio	*SS* *′*	*P* (%)
Phosphorous Level	2	261.91	130.95	76.24	258.47	17.95
Temperature (°C)	2	121.56	60.78	35.39	118.13	8.20
Surface Preparation	2	723.91	361.95	210.72	720.48	50.02
Time	2	193.78	96.89	56.41	190.34	13.21
Error	81	139.13	1.72			10.62
Total	89	1440.30				100.00

**Table 4:** Optimal values for minimum roughness.

Factor	Level Description	Level	Contribution (µm)
Phosphorous Level	Medium	2	−2.091
Temperature (°C)	High	3	−1.090
Surface Preparation	Organic	1	−3.454
Time	Low	1	−1.937
Factor Contribution			−8.572
Grand Average			16.331
Result at Optimum			7.759

**Table 5:** ANOVA of thickness.

Factor	DOF	*SS*	*V*	F-Ratio	*SS* *′*	*P* (%)
Phosphorous Level	2	76,979.40	38,489.70	1133.93	76,911.51	35.12
Temperature (°C)	2	90,718.23	45,359.11	1336.31	90,650.34	41.40
Surface Preparation	2	8530.40	4265.20	125.66	8462.51	3.87
Time	2	40,010.49	20,005.24	589.37	39,942.60	18.24
Error	81	2749.43	33.94			1.38
Total	89	218,987.95				100.00

**Table 6:** Optimal values for thickness reduction.

Factor	Level Description	Level	Contribution (µm)
Phosphorous Level	Low	1	23.72
Temperature (°C)	High	3	43.96
Surface Preparation	Chempolish	3	13.75
Time	High	3	17.34
Factor Contribution			98.77
Grand Average			50.46
Result at Optimum			149.23

## Data Availability

Data are contained within the article.
